# A large family having Muckle Wells Syndrome

**DOI:** 10.1186/1546-0096-13-S1-P31

**Published:** 2015-09-28

**Authors:** SS Kilic, S Cekic

**Affiliations:** 1 Uludag University Medical Faculty, Pediatric rheumatology, Bursa, Turkey

## Introduction

CAPS is a rare autoinflammatory disease associated with mutations in the NLRP3 gene that result in overactivation of the inflammasome, increased secretion of IL-1beta and IL-18, and systemic inflammation. Mutations in the *NLRP3* gene on chromosome 1q44 causes cryopyrinopathies. These are autosomal dominant disorders with varying penetrance, which may also present *de novo*. MWS is an intermediate phenotype characterized by chronic or intermittent episodes of fever, headache, urticarial rash, arthralgias or arthritis, CNS involvement, ocular disorders and progressive deafness. Treatment is based on IL-1 antagonism, which usually results in prompt clinical response and may prevent amyloidosis.

## Methods

Here we present a family whose 11 members have similar symptoms. Clinical data is collected during the course of ongoing patient care.

## Results

We evaluated the clinical features of 11 patients who were referred to our center. The median age of the patients was 25 years (range: 9-65 years). The ratio of females /males was 1.2 (6/5). All patients had arthritis with exacerbation on exposure to cold and ocular involvement, mostly in the form of conjunctivitis and far less commonly uveitis, irideal synechiae, band keratopathy, cataract, and impaired vision. The median age of onset of arthritis was 7 years (2-30 y), the median age of onset of ocular involvement was 8 years (2-45 y). Hearing loss in 73.6% of patients was detected. The median age of onset of hearing loss was 15 years (12-63 y). All patient except one had urticarial rash. The median age of onset of urticarial rash was 8 years (7-30 y). Genetic testing for mutations of NLRP3 gene has not done yet.

Two patients were treated with canakunimab (Ilaris, Novartis, Switzerland) which is a human anti-IL-1 beta monoclonal antibody given by subcutaneous injections every 8 weeks (2mg/kg). Following canakunimab treatment, attacks of arthritis and urticaria are getting fully under control, advances in keratopathy and hearing loss could be partially controlled.

## Discussion

MWS is characterized by recurrent fever and urticarial rash, progressive sensorineural deafness and the development of secondary amyloidosis. Treatment is based on IL-1 antagonism, which usually results in prompt clinical response and may prevent amyloidosis.

